# Deciphering angiotensin converting enzyme 2 (ACE2) inhibition dynamics: Carnosine's modulatory role in breast cancer proliferation – A clinical sciences perspective

**DOI:** 10.1016/j.heliyon.2024.e38685

**Published:** 2024-09-29

**Authors:** Sarah A. Melhem, Loai M. Saadah, Zeena S. Attallah, Iman A. Mansi, Saja H. Hamed, Wamidh H. Talib

**Affiliations:** a Department of Clinical Pharmacy, Faculty of Pharmacy, Applied Science Private University. Amman, Jordan; b Department of Clinical Pharmacy and Pharmacy Practice, Faculty of Pharmaceutical Sciences, Hashemite University, Zarqa, Jordan; c Department of Pharmaceutics and Pharmaceutical Technology, Faculty of Pharmaceutical Sciences, Hashemite University, Zarqa, Jordan; d Faculty of Allied Medical Sciences, Applied Science Private University. Amman, Jordan

**Keywords:** Carnosine, Angiotensin-converting enzyme 2 (ACE2), Inhibitor, Breast cancer, Triple negative, Luminal, MCF-7. MDA-MB-231

## Abstract

**Background:**

Angiotensin-converting enzyme 2 (ACE2) is a pivotal molecular nexus linking novel coronavirus disease to breast cancer. In-silico investigations have repurposed carnosine for both these conditions based on its potential ACE2 inhibitory properties.

**Methods:**

Utilizing an ACE2 inhibitor screening kit, we determined the inhibitory range of carnosine doses. Subsequently, we examined the effect of carnosine on ACE2 expression in supernatants from various breast cancer cell lines (MCF-7, MDA-MB-231, and EMT-6). Additionally, we compared ACE2 activity in cell line pellets with and without carnosine and a putative ACE2 activator using a fluorometric activity assay kit. Finally, we performed a 3-(4,5-dimethylthiazol-2-yl)-2,5-diphenyl-2H-tetrazolium bromide (MTT) assay across overlapping concentrations.

**Results:**

Carnosine exhibited dose-dependent ACE2 inhibition within the 100–300 mM range. ACE2 expression significantly diminished after exposure to carnosine for 2 and 24 h in MDA-MB-231 and MCF-7 cell lines, respectively. MTT assay unveiled notable antiproliferative effects in MDA-MB-231 (50 % survival at approximately 265 mM) and EMT-6 cell lines (unquantifiable 50 % survival dose). Conversely, the MCF-7 cell line displayed a modest increase in proliferation (Effective concentration 50–186 mM, ∼40 % increased survival).

**Conclusion:**

This pioneering study delineates evident dose-dependent ACE2 inhibition by carnosine. Moreover, it unveils the modulatory impact of this ACE2 inhibitor in breast cancer cell lines. Carnosine demonstrated a significant antiproliferative effect on aggressive cell lines while sparing luminal cell lines from substantial toxic or proliferative effects.

## 1 Introduction

Researchers have discerned a reciprocal interrelation between breast cancer and the novel coronavirus disease 2019 (COVID-19). Notably, COVID-19 appears to confer resistance to chemotherapy, consequently predisposing breast cancer patients to the viral infection [1]. At the molecular level, the two conditions have numerous associations. A noteworthy linkage is the higher overall disease-free survival observed in cases exhibiting elevated ACE2 expression [2]. Utilizing the TCGA TARGET GTEx study dataset, Bhari et al. demonstrated that all breast cancer subtypes generally have diminished ACE2 expression in comparison to normal breast tissue. Mechanistically, ACE2 exhibits a distinctive transcription signature concerning tumor immune infiltration (TIL) markers, encompassing pro-tumorigenic and anti-tumorigenic facets within each molecular breast cancer subtype. Intriguingly, however, ACE2 expression is substantially lower in luminal subtypes when contrasted with the more aggressive basal and Human Epidermal Growth Factor Receptor 2 (HER2) enriched cell lines [3]. Consistent with this counterintuitive finding, Nair et al. established that elevated ACE2 expression is an unfavorable prognostic marker in HER2-enriched tumors [4].

Moreover, Jiang et al. revealed a post-infection reduction in ACE2 expression following severe acute respiratory syndrome coronavirus 2 (SARS CoV-2) infection. This downregulation alters the immune infiltration levels of CD8^+^ T cells, CD4^+^ T cells, and neutrophils, exacerbating the prognosis of luminal breast cancer subtypes [5]. In summary, ACE2 activity and expression may play divergent and contrasting roles in less aggressive luminal subtypes compared to the more aggressive basal and HER2-enriched subtypes.

Redirecting focus toward the involvement of ACE2 in resistant and more aggressive breast cancer cell lines, such as HER2 and basal-like, aligns seamlessly with the research findings above. Zuo et al. eloquently showcase the specific upregulation of ACE2 within drug-resistant breast cancer cells. Their meticulous exploration further delineates that chemotherapy's induction of ACE2 expression instigates chemoresistance. This ACE2 upregulation/resistance relationship hinges upon reactive oxygen species, intricately mediated by the ROS-AKT-HIF-1α pathway. Conversely, the strategic depletion of ACE2 not only reversed resistance to Epirubicin but also concurrently fostered the proliferation of drug-resistant breast cancer cells. Additionally, ACE2 manifested robust correlations with genes associated with breast cancer resistance, with heightened ACE2 expression serving as a preceptive marker for unfavorable prognosis among patients undergoing chemotherapy [6]. Consequently, the theoretical decoupling of cytotoxic effects and ACE2 upregulation through the administration of agents inhibiting ACE2 emerges as a promising therapeutic strategy in the nuanced management of aggressive breast cancer.

Carnosine, a compound endowed with potent anticancer properties, has demonstrated remarkable efficacy across diverse cancer cell lines. In breast cancer, the luminal cell line ZR-75-1 experienced an apparent inhibition of proliferation by the fifth day when exposed to carnosine concentrations ranging from 100 to 200 mM [7]. However, the formidable challenge posed by the rapid degradation of this natural dipeptide has spurred multiple research groups to propose innovative drug delivery solutions [8,9]. Gafaar et al. for instance, exhibited a reduction in tumor size in mice through a pegylated liposomes carnosine delivery system [8]. These inventive drug delivery approaches addressed the issue of rapid degradation and led to a notable decrease in the 50 % inhibitory concentration (IC50) of carnosine. Castelletto et al. in an exemplary demonstration, illustrated that the IC50 of carnosine-derived lipidated peptide for two breast cancer cell lines, namely MCF-7 and MDA-MB-231, fell within the micromolar range [10]. Another nanoparticle carnosine delivery system consistently yielded an IC50 of approximately 60 microMolar for both MCF-7 and MDA-MB-231 cell lines [11]. In clinical settings, while carnosine remains somewhat unfamous, a phase III randomized trial elucidated its efficacy in mitigating dysphagia—the predominant clinical manifestation in breast cancer patients undergoing adjuvant radiotherapy—when complexed with zinc [12]. This complexation with zinc underscores the potential role of ACE2, a metalloenzyme with a zinc atom at its active site, inhibition. In an insightful review, Maugeri et al. analyzed the broader spectrum of carnosine's anticancer effects. According to their elegant analysis, carnosine's anticancer effects follow its regulatory influence on the expression and secretion of various cytokines and chemokines directly implicated in cancer. These include but are not limited to, enhanced expression of IL-1β, increased secretion of IL-10, GM-CSF, and TNFα, and decreased secretion of IL-8 [13].

On the other hand, other prior studies have delineated that carnosine, amid its multifaceted properties, exhibits antioxidant activity and counters reactive oxygen species (ROS). It interferes with pivotal transcription factors such as NF-κB, AP-1, p53, HIF-1α, PPAR-γ, β-catenin/Wnt, and Nrf2. These molecular interactions also relate to carnosine's cardiometabolic effects. Among these factors, HIF-1α stands out, as previously mentioned, as the conduit through which ACE2 upregulation promotes chemoresistance. Hence, by mitigating oxidative stress and chronic inflammation—established hallmarks of cancer—carnosine operates through a mechanism seemingly shared with ACE2 [14,15].

From a chemical perspective, carnosine resembles the structural scaffolds of ACE2 inhibitors, boasting an approximate 83 % similarity [16,17]. Computational investigations conducted in silico have unveiled that carnosine exhibits inhibitory interactions at the ACE2 active site and three additional pockets situated at the interface of ACE2 and the spike protein during the host cell uptake of SARS-CoV-2 [18]. Notably, carnosine forms a complex with the zinc atom at the ACE2 active site, and its inhibitory interactions rank third in preference at the four sites above. Precisely, at the ACE2 active site, carnosine orchestrates a coordinated interaction with the zinc atom, wherein the lone pair of electrons from the carboxylic acid moiety engages in sharing with the vacant orbital of the zinc atom—a pivotal characteristic in the binding of inhibitors to metalloenzymes. Furthermore, the imidazole ring within the carnosine structure engages in pi-pi stacking interactions with His401.

In contrast, carnosine's terminal primary amino group participates in a hydrogen bond with Arg514 and a pi-cation interaction with Tyr 515. Intriguingly, this study underscores a chemical kinship between carnosine and melatonin, where both molecules share an aromatic structure linked by a two-carbon bridge to an amide group, mirroring the structural features of established ACE2 inhibitors [18]. Consequently, it comes as little surprise that melatonin, akin to carnosine, modulates plasma metabolites in a triple-negative murine breast cancer xenograft model, exerting an influence on tumor progression and yielding a less aggressive tumor behavior. In summation, it is conceivable that carnosine may harbor ACE2 inhibitory properties, constituting a foundational rationale for its anticancer effects. However, it is crucial to acknowledge the absence of conclusive evidence substantiating this concept thus far. Hence, the principal objective of this study lies in validating the hypothesis that carnosine, at the very least, functions as a mild ACE2 inhibitor. Furthermore, we postulate that the mild anticancer efficacy of carnosine manifests within a concentration range that aligns with its ACE2 inhibition activity. Finally, we hypothesize that ACE2 may provide a context-specific target and thus may have varied effects on different breast cancer cell lines which can be quantified using MTT assay in these same concentration ranges.

## 2 Methods

### 2.1 Chemicals

L- Carnosine was purchased from (GE5095, Glentham Life Sciences Ltd, Corsham, United Kingdom) and stored below 25 °C until use. Carnosine was prepared by dissolving different concentrations with different solvents. Amounts ≤10 mg of Carnosine were dissolved in water, dimethyl sulfoxide (DMSO, 11 %) (CAS 67-68-5, Santa Cruz Biotechnology, Inc., Dallas, Texas, USA), appropriate media as detailed in sections below, and a mixture of DMSO - media. A mixture of DMSO and media was used to dissolve the higher concentrations. Then the solution was used to treat breast cancer cell lines (MCF-7, MDA-MB231, EMT-6) and Vero cell lines (All cell lines were taken from Applied Science Private University Cell Culture Lab, Amman, Jordan). All these cell lines were cultured with increasing concentrations of carnosine from 2 to 530 mM for 24 h for the MTT assay (Genochem world, Spain), from 7 to 442 mM for the expression experiments, and 55–442 mM for the ACE2 inhibitor kit. We selected these ranges for carnosine on the basis of previously reported ranges by other researchers [7].

### 2.2 ACE2 inhibitor screening kit

ACE2 inhibitor screening kit (ab273373, Abcam, Boston, MA, USA) was purchased and stored in the dark at −20 °C. On the day of the experiment, kit was thawed for 30 min before use. At first, a volume of 198 μL of ACE2 Dilution Buffer was introduced into the ACE2 enzyme vial. Subsequently, a working solution of ACE2 enzyme was made by adding 2 μL of the diluted ACE2 enzyme to 48 μL of the ACE2 assay buffer (final volume of 50 μL). The solution was stirred extensively and thereafter dispensed 50 μL of the resultant mixture into each of the designated wells of a 96-well microtiter plate. Following that, Enzyme Control (solvent dimethyl sulfoxide or DMSO), Screening Compound (i.e., carnosine), and standard kit Inhibitor Control were formulated by dissolving potential inhibitors in a buffer solution at concentrations that were 10 (DMSO) to 100 times (carnosine & standard kit inhibitor) greater than the eventual test concentration. To attain a 10-fold dilution of the designated test concentration, the solution was diluted using Assay Buffer XI/ACE2 Assay Buffer. Then, 10 μL of test inhibitors (carnosine), standard kit inhibitor and enzyme control (DMSO) were added to each designated well which also contained 40 μL substrate mix and 50 μL of assay buffer. The samples underwent incubation at ambient temperature for a duration of 15 min before addition to the wells. A volume of 40 μl of the Substrate Mix, prepared with 2 μL of ACE2 substrate and 38 μL of assay buffer, was added to each well. The combination was fully blended and thereafter, the fluorescence intensity was observed in kinetic mode for a period of 1 h at room temperature. Specifically, the fluorescence intensity was measured at 2, 17, 32, 47, and 62 min using BioTek Cytation 5 Cell Imaging Multimode Reader (Agilent Technologies, Inc. Headquarters, CA, USA). The excitation and emission wavelengths employed were 320 nm and 420 nm, correspondingly. The slope for each well was determined by studying the linear portion of the fluorescence plot. The evaluation of ACE2 inhibition was performed across a concentration range of 12.5–100 mg/ml (equivalent to ∼ 55.2–442 mM). This was determined on the basis of previously reported research [7]. We have gone a little above (∼double) and below (∼half) these ranges. The molar mass of carnosine is 226.3 g/mol. For conversion between concentrations in mg/ml and millimolar (mM) we used the following formula:

ConcentrationinmM=(Concentrationinmg/ml)/(Molecularweightinmg/mmol)X1000

Each of carnosine, standard kit inhibitor, and DMSO were tested in triplicates at four dilutions (concentrations). For carnosine these were approximately 55, 110, 221, and 442 mM. For the standard kit inhibitor concentrations were 6.25, 12.5, 25 and 50 nM. These concentrations are per the protocol of the kit. For DMSO, it was four concentrations matching to those used with carnosine; namely, 0.0625 %, 0.125 %, 0.25 %, and 0.5 %. These were scaled to the kit standard inhibitor range to make it easier to visualize the results. Subsequently, the % activity was calculated for each of the concentrations in one of two ways; either by dividing the slope of the fluorescence line for each measurement by the slope of the matching fluorescence line slope for enzyme control (% activity 1), or by dividing by the average slope for all the enzyme controls fluorescence lines (% activity 2). Finally, % inhibition was calculated for each concentration and measurement by the following equation:

%Inhibition=1−%Activity

Then we used IBM SPSS Statistics (version 26.0.0.0) to calculate the 95 % confidence interval for the triplicates at each concentration and for each drug or solvent. For comparisons of the drugs, overlapping means and 95 % confidence intervals were deemed statistically insignificant differences, whereas non-overlapping means and 95 % confidence intervals were a clear proof of statistically significant difference.

### 2.3 ACE2 expression kits

These are ELISA kits which operate by utilizing the Sandwich-ELISA method, employing a Micro Elisa strip plate precoated with antibodies specific to ACE2. Samples or standards are introduced into designated wells, followed by the addition of ACE2-specific Horseradish Peroxidase (HRP)-conjugated antibodies, allowing for incubation after each addition step. Excess materials are removed by washing, and the 3,3',5,5'-Tetramethylbenzidine (TMB) substrate solution is then added to detect the presence of peroxidase activity (solution turn to blue). Finally, positive wells exhibit a color change to yellow upon the addition of an acidic stop solution. The optical density (OD) is subsequently measured at a wavelength of 450 nm using a spectrophotometer, establishing a direct relationship between ACE2 concentration and OD, enabling the determination of ACE2 levels in the samples. The actual sources of these kits are mentioned in the respective sections below.

In the preparation of carnosine, 100 mg are accurately measured and dissolved in DMSO under sterile conditions to form a solution with the needed concentration. Serial dilutions are then made to prepare a range of exposure concentrations from 7 mM to 442 mM. For cell culture preparation, MCF-7 cells are thawed and cultured according to established protocols in Roswell Park Memorial Institute (RPMI) medium supplemented with 10 % Fetal Bovine Serum (FBS, ECS0180L, Euroclone, Milan, Italy) and 1 % penicillin-streptomycin solution (15-140-122, Thermo Fisher Scientific, Waltham, MA, USA). These cells undergo regular monitoring for growth and are sub cultured as necessary. Collection of MCF-7 cells is facilitated by trypsin-EDTA which help in detaching and dissociating adherent cells, and their concentration is adjusted to achieve a count of 10,000 cells. The cells are then evenly distributed among 25 ml cell culture flasks, with carnosine solution (prepared earlier) added to two of them. After careful mixing, the cells are cultured at 37 °C in a carbon dioxide incubator for specified durations. A control flask without carnosine treatment is also maintained. Following incubation, cells are harvested using trypsin-EDTA, diluted in Phosphate-Buffered Saline (PBS), and subjected to repeated freezing and thawing to release intracellular components. The supernatant is collected after centrifugation.

Similarly, MDA-MB-231 cells undergo the same procedures, albeit cultured in Dulbecco's Modified Eagle's Medium (DMEM) (Hyclone, SH30022. LS, Shanghai, China) with the same supplements. The steps for cell collection, concentration adjustment, carnosine treatment, incubation, and intracellular component extraction are repeated as outlined for MCF-7 cells. These rigorous procedures ensure the accurate handling of both cell lines for subsequent analysis.

### 2.4 ACE2 expression in murine breast cancer cell line

This experiment investigated the effects of different concentrations of carnosine on mouse ACE2 expression. Purchased and stored at 2–8 °C until date of use, mouse Angiotensin Converting Enzyme 2 ELISA kit (SL0973Mo, Sunlong Biotech Co. LTD, Hangzhou City, Zhejiang, China) was utilized in this part. Carried out at the Applied Science Private University (ASU) – Faculty of Pharmacy (FOP), the study examined concentrations ranging from 28 to 442 mM, with triplicate measurements at 2 h and 24 h post-exposure. Five standard ACE2 concentrations were prepared from Standard No. 1 (720 pg/ml) to Standard No. 5 (60 pg/ml). Initially, 300 μL of the Original Standard containing 1080 pg/ml was mixed with 150 μL of Standard diluent in a small tube, yielding Standard No.1 with a concentration of 720 pg/ml. Subsequently, Standards No. 2 to No. 5 were prepared by sequentially diluting the preceding standard with the diluent. Each standard received 150 μL of diluent after extracting 150–300 μL from the previous standard. The standards were labeled with distinct letter combinations (e.g., A1, A2, B1, B2) and distributed into ten wells of a microplate, with 50 μL of each standard aliquoted into two wells. In the Micro Elisa strip plate, one duplicate well was designated as a blank control. In each sample well, 40 μL of sample dilution buffer and 10 μL of mouse cell samples (EMT-6 treated with carnosine for 24 h, EMT-6 treated with carnosine for 2 h, or untreated EMT-6 cells) were added, with a dilution factor of 5. The cells were carefully placed at the bottom of the well to avoid contact with the walls, and the contents were gently mixed. The plate was sealed with a Closure plate membrane and incubated at 37 °C for 30 min. The highly concentrated washing buffer was diluted with distilled water at a ratio of 30:1. After removing the Closure plate membrane, the contents were aspirated and replenished with the diluted washing solution, followed by five consecutive washing steps. Next, 50 μL of HRP-Conjugate reagent were dispensed into each well except the blank control. The plate underwent another round of incubation and washing. Then, 50 μL of Chromogen Solution A and 50 μL of Chromogen Solution B were added to each well, gently mixed, and incubated at 37 °C for 15 min in darkness. The reaction was stopped by adding 50 μL of stop solution to each well, resulting in a color change from blue to yellow. Finally, the absorbance optical density (O.D.) was measured at a wavelength of 450 nm using an ELISA microplate reader (BioTek, Winooski, VT, USA).

### 2.5 ACE2 expression in human cell lines

A similar protocol was followed for the human ACE-2 ELISA kit method. It was performed using human Angiotensin Converting Enzyme 2 ELISA kit (SL2129Hu, Sunlong Biotech Co. LTD, Hangzhou City, Zhejiang, China). Kit items were stored appropriately at 2–8 °C. Similarly, the kit contained 1 Microelisa stripplate, 2 Closure plate membranes, 1 Standard (270 pg/ml, 0.5ml × 1 bottle), 1 Standard diluent (1.5ml × 1 bottle), 1 HRP-Conjugate reagent (6ml × 1 bottle), 1 Sample diluent (6ml × 1 bottle), 1 Chromogen Solution A (6ml × 1 bottle), 1 Chromogen Solution B (6ml × 1 bottle), 1 Stop Solution (6ml × 1 bottle), and 1 wash solution (20 ml (30X) × 1bottle). Carried out at the ASU – FOP, the study examined concentrations ranging from 7 to 442 mM, with triplicate measurements at 2 h and 24 h post-exposure. Five standard ACE2 concentrations were prepared from Standard No. 1 (180 pg/ml) to Standard No. 5 (15 pg/ml). Initially, 300 μL of the Original Standard at a concentration of 270 pg/ml were mixed with 150 μL of Standard diluents in a small tube to produce Standard No.1 with a concentration of 180 pg/ml. Sequentially, Standards No.2 to No.5 were prepared by transferring 150–300 μL from the preceding standard and supplementing each with 150 μL of diluents. Each standard was then labeled with distinct letter labels (e.g., A1, A2, B1, B2). Ten wells were arranged in a microplate, with one duplicate well designated as a blank control left empty. Subsequently, 50 μL of each diluted standard were dispensed into two wells using a pipette. For samples, in each well, 40 μL of sample dilution buffer and 10 μL of human cell samples, treated with carnosine for varying durations or left untreated, were added, with a dilution factor of 5 applied. Care was taken to ensure the cells settled at the well bottom without contacting the walls. Gentle agitation was employed for thorough mixing. The microplate was sealed with a Closure plate membrane and incubated at 37 °C for 30 min. Washing buffer was diluted with distilled water at a ratio of 30:1 before each washing cycle, conducted five times. HRP-Conjugate reagent was dispensed into each well except the blank control, followed by repeated incubation and washing steps. Chromogen Solution A and B were added to each well, gently mixed, and incubated at 37 °C for 15 min in the absence of light to avoid sample exposure. The reaction was stopped by adding stop solution, resulting in a color transition from blue to yellow. Finally, the absorbance optical density (O.D.) was measured at a wavelength of 450 nm using an ELISA microplate reader (BioTek, Winooski, VT, USA).

### 2.6 Cell line, subculture, and cellular conditions

In evaluating the diverse impacts of carnosine, various breast cancer cell lines, such as MCF-7 and MDA-MB-231, EMT-6, and normal cells (Vero) (ASU-FOP internal cell bank, Amman, Jordan) were employed. To maintain optimal cell growth conditions, a complete tissue culture medium was utilized in conjunction with appropriate incubation settings, including a temperature of 37 °C, 5 % CO_2_, and 95 % humidity. The comprehensive tissue culture medium was formulated by adding 10 % fetal bovine serum (ECS0180L, Euroclone, Milan, Italy), 1 % L-Glutamine (SH30034.01, Cytiva, Marlborough, MA, USA), 1 % Penicillin-Streptomycin solution (15-140-122, Thermo Fisher Scientific, Waltham, MA, USA), 0.1 % Gentamycin sulfate solution (L0012, Biowest, Nuaillé, France.), and 0.1 % Non-Essential Amino acids (X0557, Biowest, Nuaillé, France). The initial step involved thawing the selected cell lines (MDA-MBA-231, MCF-7, EMT-6, and Vero-normal cells) from liquid nitrogen in a 37 °C water bath. Subsequently, the cells were promptly transferred into a 25 ml tissue culture flask containing 15 ml of the complete tissue culture medium specific to the respective cell line. Essential information, including the cell line name, date of culture, and batch number, was recorded on the culture flask. The cells were then incubated and monitored daily under the microscope until reaching their desired confluence.

In breast cancer research, the triple-negative MDA-MB-231 and estrogen receptor-positive MCF-7 cell lines are widely employed. The cells were thawed, washed twice, and suspended in FBS- and antibiotic-supplemented DMEM medium before investigation. To achieve enough completely functioning cells, the cells were grown at 37 °C with 5 % CO_2_ for 4–5 rounds. Thus, only 4th and 5th passage cells were used in the study. Carboxyfluorescein succinimidyl ester (CFSE, C34554, Sigma-Aldrich, Saint Louis, MO, USA) was used to label MDA-MB-231 and MCF-7 cells before the experiment. Staining was done to check for cell proliferation following co-culture.

In 25 cm³ (VWR) flasks, MCF-7 and MDA-MB-231 cell lines were cultured in appropriate media as mentioned above, at 37 °C in a humidified incubator with 5 % CO_2_. MDA-MB-231 cells were sub cultured for 24 h and MCF-7 for 1 week. The adhering cells were rinsed with 2 mL of Phosphate Buffered Saline (PBS, ECS8103, Euroclone, Milan Italy) after aspirating the media. To detach cells, 2 ml of trypsin (12330, Biowest, Nuaillé, France) was applied and incubated at 37 °C for 3 min. The cell density (cells/mL) was calculated using Trypan blue dye (ECS8003, Euroclone, Milan, Italy) and counting chamber. The percentage of viable cells over the total number of viable cells added to dead cells was used to calculate cell viability (%), which was tested frequently to ensure they were healthy. After adding media to a 96-well plate using a multichannel pipette and incubation at 37 °C, the calculated number of cells was transferred. When the confluence of the cells inside the flask reached (80–90 %), trypsin/EDTA and PBS were used to separate and re-suspend the adhering cells from the flask wall, after that the flask was incubated for 2–3 min, washed with 5 ml of complete tissue culture media and placed into a sterile centrifuge tube and centrifuged for 5 min at 2000 rpm (358 G) and 4 °C. Following centrifugation, a pellet, and a supernatant was formed; the supernatant was eliminated while the pellet was re-suspended in 5 ml of tissue culture medium to form a cell suspension. Trypan blue dye was used to count cell density. The percentage of viable cells over the total number of viable cells added to dead cells was used to calculate cell viability (%), which was tested frequently to ensure they were healthy. After adding media to a 96-well plate using a multichannel pipette and incubation at 37 °C, the calculated number of cells was transferred.

### 2.7 Antiproliferative MTT assay

Each cell line underwent rapid culturing and trypsinization to initiate seeding into a 96-well tissue culture plate, with 10,000 cells/well for each line. After an incubation period of approximately 24 h, the media in each well were replaced, and the adherent cells were treated in triplicate with decreasing concentrations of Carnosine via serial dilution (520–2 mM). An additional 100 μl of media was added to each well, resulting in a total volume of 200μl/well, and the cells were left to incubate for about 48 h. Cell viability was determined using the MTT (3-(4,5-dimethylthiazol-2-yl)-2,5-diphenyltetrazolium bromide) assay (G5431, Promega, Madison, WI, USA). This colorimetric reduction method measured changes in mitochondrial activity by enzymatically converting the tetrazolium salt MTT into formazan crystals. The quantification of viable cell numbers was achieved by assessing formazan concentration, indicated by the optical density (OD) measured at 550 nm using ELISA microplate reader (BioTek, Winooski, VT, USA). Optical density was compared to control optical density for each measurement, allowing the calculation of the average % survival for the three triplicates. A range of carnosine concentrations, up to a maximum of 120 mg/ml (530 mM), was used during the 24-h exposure of all culture cell lines before reading optical density.

### 2.8 Statistical analyses

All in vitro experiments in this study were done in triplicates. We have used continuous endpoint, two independent sample study equations to calculate the needed sample size for every experiment. An example is provided here; to detect a difference in expression (fluorescence) at a comparable dilution for carnosine and DMSO of 1600 and 2200, respectively, and assuming the standard deviation of measurements would be 250, we would need 3 measurements in each group giving us a power of 80 % to detect a statistically significant difference at the 0.05 alpha level of significance. IBM SPSS Statistics 26.0 was used to graph data and generate error bars with 95 % confidence intervals (95 % CI) and/or standard errors of the mean (SEM). Effective concentration (EC50) and inhibitory concentration (IC50) were calculated using the four-parameter sigmoidal equation at the following pages (URL: https://www.aatbio.com/tools/ec50-calculator) and (URl: https://www.aatbio.com/tools/ec50-calculator), respectively, both accessed on Tuesday, December 5th, 2023). EC50 represents the concentration resulting in a 50 % increase in breast cancer cell line % survival, while IC50 indicates the concentration causing a 50 % reduction in breast cancer cell line % survival. MTT survival sigmoidal curve graphs were generated using the application MyCurveFit (Beta Version, available online at URL: https://mycurvefit.com/, accessed Thursday, February 29th^,^ 2024). 95 % CI or SEM error bars for a group mean overlapping with another group mean was reasonably considered a clear proof of statistically insignificant differences.

## 3 Results

### 3.1 Carnosine and ACE2 inhibition

Carnosine's impact on ACE2 inhibition was both conspicuous and dose-dependent within the examined spectrum spanning from 55 to 442 mM. However, this inhibition exhibited a tempered character when juxtaposed with a standard inhibitor. This standard inhibitor demonstrated a more potent ACE2 inhibitory effect within a markedly lower concentration range, specifically ranging from 6.25 to 50 nM. The computation of % inhibition for each concentration necessitates the division of the slope derived from the meticulously fitted linear equation characterizing fluorescence across five temporal points for each concentration by the commensurate average slope associated with the enzyme control (DMSO) as delineated in Table 1.

**Table 1:** Inhibition kit fluorescence detected at Ex/Em = 320/420 nm for the different time points in Mean ± Standard Deviation with Linear Trendline Slope^a^ and % Inhibition Average^b^ below.

Time Point in Minutes					
Test Compound	2	17	32	47	62
Carnosine (mM)					
442	622 ± 28	713 ± 55	762 ± 50	897 ± 57	896 ± 143
				Slope = 4.9	% Inhibition = 85 %
221	1549 ± 141	1891 ± 292	2386 ± 135	2319 ± 280	3036 ± 119
				Slope = 22.7	% Inhibition = 29 %
110	1856 ± 243	2469 ± 211	2868 ± 255	3291 ± 425	3402 ± 540
				Slope = 26.1	% Inhibition = 18 %
55	1948 ± 516	2591 ± 54	2741 ± 449	3588 ± 530	3777 ± 349
				Slope = 31.0	% Inhibition = 3 %
Kit Inhibitor (nM)					
50	972 ± 187	833 ± 116	952 ± 31	1027 ± 140	1072 ± 130
				Slope = 2.6	% Inhibition = 92 %
25	1524 ± 302	2109 ± 153	2087 ± 419	2380 ± 337	2595 ± 223
				Slope 16.1	% Inhibition = 50 %
12.5	1651 ± 243	2131 ± 145	2388 ± 117	2934 ± 117	3176 ± 338
				Slope = 25.7	% Inhibition = 20 %
6.25	1875 ± 95	2608 ± 210	3097 ± 69	3833 ± 648	3987 ± 271
				Slope 36.3	% Inhibition = 0 %
Enzyme/DMSO Control^c^					
Dilution 1	1638 ± 46	2236 ± 119	2310 ± 332	2949 ± 197	3545 ± 386
				Slope = 30.2	% Inhibition = 5 %
Dilution 2	2129 ± 169	2560 ± 368	3155 ± 95	3395 ± 463	3935 ± 261
				Slope = 29.7	% Inhibition = 7 %
Dilution 3	1726 ± 59	2212 ± 402	2741 ± 158	3320 ± 158	3469 ± 241
				Slope = 30.6	% Inhibition = 4 %
Dilution 4	2013 ± 186	2618 ± 210	3140 ± 147	3788 ± 274	4220 ± 473
				Slope 37.2	% Inhibition = 0 %

a Measurement Sample Size (N) = 3 for each time point, x is the time point and y is the fluorescence reading, actual slopes and intercepts were divided by 1000 and rounded to the nearest integer.

b %Inhibition was calculated from %activity which was the fraction of the trendline slope to the matching enzyme control dilution slope.

c DMSO is the solvent dimethyl sulfoxide used as a control.

**Fig. 1:**
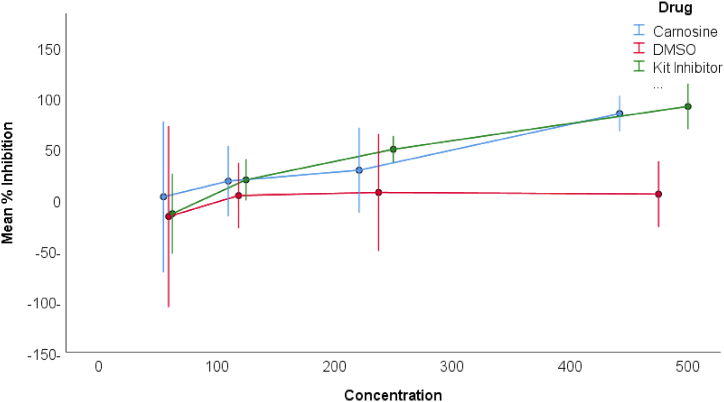
Mean % ACE2 Inhibition for carnosine, standard kit inhibitor, and DMSO. Error bars represent 95 % confidence interval of the mean of three measurements for each data point. **Note:** X-axis units are as follows: 100 on the X-axis scale = 100 mM for carnosine, 10 nM of standard kit inhibitor, and 0.105 % of DMSO.

The pinnacle of ACE2 inhibition, ascribed to carnosine, reached 85 % at the concentration of 442 mM. In stark contrast, the standard inhibitor achieved a maximum of 92 % ACE2 inhibition, notably at the concentration of 50 nM, as artfully illustrated in Fig. 1. It is noteworthy that disparities in % ACE2 inhibition attained statistical significance solely at the loftiest concentration for both carnosine and the standard ACE2 kit inhibitor. Notwithstanding, meticulous pairwise comparisons between the two compounds demonstrated statistical insignificance across all corresponding concentrations.

Carnosine emerges as approximately 100k times less potent than its standard ACE2 kit inhibitor counterpart. Furthermore, it is intriguing to note that carnosine, in stark contradistinction to a presumed ACE2 activator (data not shown), exhibited ACE2 inhibition across both breast cancer cell lines—namely MCF-7, MDA-MB-231, and EMT-6. This discerning observation was gleaned from an activity kit employed within a concomitant research endeavor conducted by a parallel investigative group, albeit the specific data remains regrettably undisclosed at this juncture.

### 3.2 Effect of carnosine on ACE2 expression levels

The derivation of ACE2 standard expression curves yielded robust regression equations, attesting to the precision of the model fit. For the human kit, the final regression equation for ACE2 concentrations ranging from 0 to 180 pg/ml is eloquently expressed as:

$$y=3.623989-3.231878*\hat{e}\left(-0.135636*x\right),\text{Regression}\;r2=0.99$$

where y is the absorbance or optical density at 450 nm and x is the matching ACE2 concentration.

This equation signifies an impeccable conformity to the observed data. This equation serves as a powerful tool for the computation of baseline ACE2 expression levels within human breast cancer cell lines, specifically MCF-7 and MDA-MB-231, both in their unaltered states and after exposure to carnosine for durations of 2 or 24 h.

Similarly, for the mouse kit, the derived regression equation for ACE2 concentrations spanning from 0 to 720 pg/ml assumes an elegant form:

$$y=3.668-3.464667*\hat{e}\left(-1*x\right),\text{Regression}\;r2=0.98$$

where y, again, is the absorbance or optical density at 450 nm and x is the matching ACE2 concentration.

This equation, exhibiting a near-perfect fit, facilitates the computation of baseline ACE2 expression levels in the EMT-6 breast cancer cell line, as well as the expression alterations following exposure to carnosine over durations ranging from 2 to 24 h.

The ensuing ACE2 expression data, meticulously tabulated in Table 2, reveals nuanced observations. Notably, MDA-MB-231 exhibits a marginally higher baseline expression (approximately 180 pg/ml) compared to MCF-7 (roughly 120 pg/ml). Conversely, ACE2 expression in the mouse cell line remains markedly low, estimated well below 60 pg/ml. A striking temporal dichotomy emerges in the response of MCF-7 to carnosine exposure, evidenced by an initial reduction in expression at 2 h, followed by a resilient rebound to baseline values at 24 h. In contrast, MDA-MB-231 experiences moderate reductions in ACE2 expression levels at 2 h for concentrations equal to or exceeding 25 mM, with incomplete restoration to baseline values. Remarkably, EMT-6 exhibits substantial initial reductions in expression at 2 h, followed by a pronounced resurgence to baseline values at the 24-h mark.

**Table 2:** ACE2 levels expressed in absorbance or optical density (OD) for all breast cancer cell lines and following 2 and 24 h of exposure to various concentrations of carnosine.

	Exposure Duration (Time of Expression Check)		
Cell Line	Test Condition^a^	2 Hour	24 Hour^b^
MCF-7	442	2.38 ± 0.17	3.68 ± 0.15
	221	1.51 ± 0.14	3.79 ± 0.12
	110	1.51 ± 0.07	3.57 ± 0.06
	55	1.40 ± 0.07	3.60 ± 0.11
	28	3.79 ± 0.06	–
	14	3.72 ± 0.11	–
	7	3.46 ± 0.10	–
	Cell Line	3.66 ± 0.04	3.67 ± 0.04
	Background	0.22 ± 0.04	0.23 ± 0.05
MDA-MB-231	442	2.05 ± 0.07	3.75 ± 0.05
	221	2.08 ± 0.04	3.07 ± 0.08
	110	2.34 ± 0.11	3.30 ± 0.08
	55	3.68 ± 0.04	3.44 ± 0.03
	28	3.73 ± 0.12	3.67 ± 0.09
	Cell Line	3.74 ± 0.27	3.76 ± 0.08
	Background	0.37 ± 0.09	0.40 ± 0.10
EMT-6	442	1.72 ± 0.04	3.32 ± 0.04
	221	1.70 ± 0.16	2.58 ± 0.13
	110	1.53 ± 0.05	2.30 ± 0.17
	55	1.68 ± 0.09	1.50 ± 0.11
	28	1.58 ± 0.14	1.04 ± 0.05
	Cell Line	3.06 ± 0.08	3.03 ± 0.14
	Background	0.26 ± 0.12	0.13 ± 0.03

a Test conditions were carnosine in the given concentrations from 7 to 442 mM or the cell line or background level of ACE2 expression.

b There were no readings or measurements for MCF-7 at the lowest three concentrations.

### 3.3 Carnosine antiproliferative effect

**Fig. 2:**
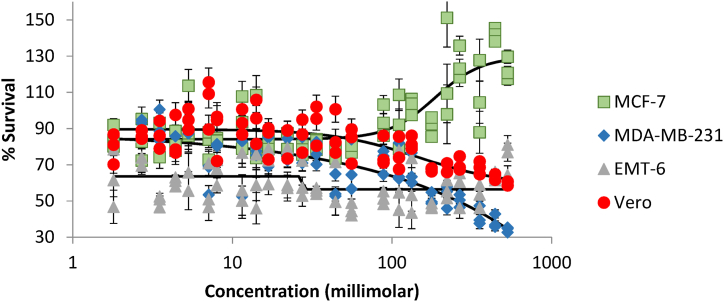
Mean % survival in MTT assay at carnosine concentrations ranging from 1.8 to 530 mM for four different cell lines. Error bars represent standard error of the mean for three measurements at each data point. **Note:** Red color (circles) is for the Vero Cell line, Green (squares) for the luminal or hormonal breast cancer cell line (MCF-7), Blue (diamond) for human triple negative breast cancer cell line (MDA-MB-231), Grey (triangle) for the murine cell line (EMT-6).

Carnosine, under scrutiny through MTT assays across diverse cell lines, unveiled disparate effects on % survival, as thoroughly documented in Table 3. As anticipated, the luminal breast cancer cell line (MCF-7) exhibited a discernible augmentation of about 40 % in survival within the 100–500 mM range of carnosine concentrations, a trend elegantly illustrated in Fig. 2. EC50 was around 186 mM for MCF-7. In marked contrast, the aggressive breast cancer cell lines, MDA-MB-231 and EMT-6, bore witness to a net antiproliferative impact. Specifically, the MDA-MB-231 cell line experienced a notable 50 % reduction in survival within this concentration range, although the overall % reduction surpassed this value. IC50 was about 265 mM for MDA-MB-231. Conversely, the EMT-6 cell line demonstrated a reduction in % survival; however, this effect remained relatively stable within the 100–500 mM concentration range and the IC50 could not be quantified. Notably, the Vero cell line displayed a modest reduction of less than 20 % in survival within the same concentration range. This nuanced response underscores the varying sensitivities of distinct cell lines to carnosine exposure in the same range of concentrations where ACE2 inhibition occurs.

**Table 3:** % Survival in MTT assay for the different cell lines at carnosine concentrations ranging from ∼2 mM to ∼530 mM (Mean ± Standard Deviation).

Concentration	MCF-7	MDA-MB-231	EMT-6	Vero
1.8	85.3 ± 6.1	80.4 ± 0.1	62.3 ± 15.9	79.4 ± 8.4
2.7	83.5 ± 11.5	92.2 ± 3.2	76.3 ± 8.3	86.4 ± 2.4
3.5	86.9 ± 10.9	90.8 ± 8.9	50 ± 3.1	86.5 ± 7.7
4.4	82.5 ± 5.6	80.3 ± 4.7	60.8 ± 2.5	84.3 ± 11.5
5.3	95.8 ± 15.5	94.1 ± 6.9	70.8 ± 18.2	95 ± 5.8
7.1	81.6 ± 7.8	63.9 ± 9.0	56.2 ± 12.9	104.8 ± 13.5
8.0	83.8 ± 0.8	77.6 ± 5.9	66.4 ± 7.2	87.8 ± 13.6
11.5	93.5 ± 14.4	76 ± 20.9	60.9 ± 13.9	93.5 ± 7.0
14.1	87.2 ± 18.6	77.5 ± 2.2	60.2 ± 14.9	94.4 ± 12.2
16.8	81.3 ± 3.0	75.9 ± 8.2	70.3 ± 10.0	83.7 ± 9.3
22.1	81.3 ± 5.4	77.4 ± 6.3	65.1 ± 12.4	83.8 ± 8.9
27.4	78.3 ± 2.5	77.2 ± 7.3	60.8 ± 10.2	86.2 ± 9.1
33.6	82.0 ± 3.2	76.2 ± 6.2	55.6 ± 1.7	92.7 ± 11.0
44.2	76.0 ± 2.6	57.2 ± 6.8	56.9 ± 12.7	86.7 ± 12.5
55.7	79.6 ± 2.4	68.4 ± 14.1	45.2 ± 3.6	81.9 ± 9.9
88.4	94.2 ± 8.7	72.4 ± 6.8	51.7 ± 3.5	76.7 ± 8.2
110.5	93.2 ± 15.0	69.3 ± 11.2	59.3 ± 16.9	75.7 ± 9.1
132.6	102.6 ± 4.5	66.8 ± 8.6	56.9 ± 15.4	82.1 ± 3.6
176.8	91.4 ± 5.4	51 ± 3.4	47.2 ± 1.4	67.3 ± 0.9
220.9	119.5 ± 28.0	53 ± 6.2	56.2 ± 5.0	67.6 ± 2.8
265.1	125.8 ± 9.0	50.2 ± 3.5	57.4 ± 10.0	70.2 ± 3.9
353.5	106.8 ± 20.0	41.5 ± 5.2	52.9 ± 6.4	68.4 ± 3.6
441.9	141.9 ± 3.7	38.5 ± 3.9	63.5 ± 2.4	62.7 ± 1.8
530.3	122.6 ± 6.3	34.3 ± 1.4	74.2 ± 8.7	59.3 ± 1.3

## 4 Discussion

This study is the first to experimentally validate that carnosine is a mild ACE2 inhibitor. It comes as a confirmation of our previous in silico findings [18]. This ACE2 inhibition is dose dependent at 55–442 mM, the same range where other researchers have demonstrated carnosine various antioxidant, anti-inflammatory and anticancer properties [7]. This mild inhibition is substantiated by the fact that carnosine is an easily hydrolyzed dipeptide [8,9]. Reformulating carnosine into nano-delivery systems resulted in more than a 1000 times improvement in its potency [10]. Therefore, one hypothesis generated from these findings is that ACE2 inhibition would also increase by a factor of 1000 with these nano formulations.

Based on our previous in silico study, we have designed a new series of novel ACE2 inhibitor compounds [19]. Our team succeeded in synthesizing, characterizing, and experimentally validating one of these compounds; namely LMS1007 (data yet to be published). LMS1007 was about 100 times more potent ACE2 inhibitor than carnosine and can also be carried in novel nano formulations with the potential of producing very strong ACE2 inhibition in the nano-to micromolar range. Therefore, future research will focus on confirming ACE2 role in aggressive breast cancer cell lines proliferation and we expect to develop a breakthrough treatment for these in the near future.

Previous research has all shown carnosine anticancer effects occur in the same range of ACE2 inhibition we found in the current study including breast, prostate, and colorectal among other cancers [13,20,21]. Moreover, Morsy et al. was consistent that carnosine has weaker potentiation effects for doxorubicin-induced cytotoxicity at the micromolar range in resistant cancer cells [22]. So again, our findings and those of others suggest a link between ACE2 inhibition in the millimolar range and anticancer effect in various cancers and therefore with the proper delivery systems carnosine anticancer effects can be employed in the management of many cancers as well as in resistant forms of cancer. Another important point is that ACE2 is a pretty much conserved protein and therefore, the pathological paths it is involved in are amenable for cross species studies, investigations, and validations.

Our study also has for the first time shown that carnosine possesses the ability to change ACE2 expression in the tested breast cancer cell lines. This concept has been minimally investigated previously. However, carnosine seems to increase the expression of Nrf2 by a mechanism which seems to be shared by ACE2 [14,15]. This has also been proposed by other research groups that have shown that carnosine may affect the expression and activity of mTOR, another signaling marker in this same pathway [23]. We have reported the findings of other research groups that linked ACE2 expression in various breast cancer cell lines to their proliferation potential [[1], [2], [3], [4]]. Additionally, downregulation of ACE2 expression was linked to worse prognosis of luminal cancer cells, whereas heightened expression conferred more cancer cell chemoresistance in more aggressive cell lines [5,6]. However, this concept of increasing proliferation of a luminal breast cancer cell line was not reported in former in vitro studies. Notably, carnosine increased the proliferation of a cell line in a study, through the AKT pathway in pigs, which was utilized to grow muscles in pigs, even though ACE2 was absent in their investigations [24]. Also, it was shown in another study on yeasts that carnosine can have both inhibitory and excitatory effects on the growth of yeasts depending on the conditions of the media as well [25]. Therefore, our study comes as the first, in this same line, to show that through ACE2 modulation, carnosine can affect cancer cell proliferation in ways that may change from one cell line to another. ACE2 expression starts high in a normal cell, then through an unknown mechanism ACE2 is downregulated and this seems to be the cells response to reduce proliferation initially. This ability is finally lost when ACE2 concentrations reach a bare minimum which is then followed by activation/upregulation of ACE2 and more proliferation and metastasis [[1], [2], [3], [4]]. In our study, ACE2 expression was minimally affected by the lower concentrations of carnosine in the luminal breast cancer cell line, MCF-7. However, there was a marked reduction in the other two aggressive breast cancer cell lines; namely MDA-MB-231 and EMT-6, followed by a resurgence in expression. This could be related to the consistent carnosine cytotoxic effects seen in these two cell lines. However, more studies are needed, with more cell lines, to fully characterize these mechanistic pathways and their contribution to cancer metastasis.

Taking the MTT assay results in this context, our findings contrast to previous research which showed reduction in % survival in luminal breast cancer cell lines in the tested millimolar range of concentrations [7,11]. This study for the first time demonstrated that ACE2 inhibition in MCF-7, a luminal breast cancer cell line, in fact may have a pro-proliferative effect, albeit small at around 40 %. Both clinical and basic science implications of these findings are paramount. On the clinical side, modulating ACE2 by inhibition may in fact be harmful at least in some luminal breast cancer cell lines. Activation on the other hand was even more problematic in our twin research study (data not shown). However, ACE2 modulation was actually consistently beneficial in the more aggressive breast cancer cell lines and may prove, in further clinical studies, a very promising strategy to control metastasis and improve the clinical outcomes. This is also a very important finding for all future research in the cancer cell line lab as controlling ACE2 may prove essential in these basic science studies. However, these findings must be confirmed with other ACE2 modulator compounds.

In conclusion, this study offers very promising findings for a modulatory effect of ACE2 on breast cancer cell lines proliferation. Initially, near normal-like cancer cells downregulate ACE2 in an effort to control proliferation where ACE2 seems to act as a balancing enzyme. Later, however, this protein seems to lose its balancing effect and ACE2 modulation becomes a major target for controlling proliferation of aggressive breast cancer cell lines. This is a very promising target and treatment strategy for breast cancer as well as other forms of chemo resistant cancers. Future research must further evaluate these findings across the board from in vitro, to animal models, all the way up to randomized controlled clinical trials in breast as well as other forms of cancer. Finally, carnosine is proven for the first time as an ACE2 inhibitor and this may well be the mechanism by which it possesses anticancer effects. More studies are needed to confirm these findings on carnosine and other ACE2 inhibitors and activators. For example, it would be a great research proposal to confirm these findings on potent ACE2 inhibitors with IC50 in the nanomolar range such as ORE-1001 (also known as MLN = 4760).

### 4.1 Limitations of the study

The concept of the study is novel. The major challenge is confirming the findings in more breast cancer cell lines with varying degrees of ACE2 expression levels. Moreover, if the concept of modulating ACE2 has a role on the management of COVID-19 is yet to be proven. However, another research team under the supervision of the corresponding author was able to discover, synthesize, characterize and experimentally validate this concept in COVID-19 on a novel molecule; namely, LMS1007. LMS1007 is 100 times more potent as ACE2 inhibitor than carnosine. In the same range of its ACE2 inhibition, we were able again to see completely superimposed inhibition of the Severe Acute Respiratory Syndrome Coronavirus 2 (SARS CoV-2) spike protein-ACE2 interaction. These results regard the new molecule LMS1007 are now under publication in a top journal in drug discovery. We have registered a patent for LMS1007 and other related compounds at the World Intellectual Property Organization (WIPO) with the following citation: reference number 19. Saadah, L., Abu Deiab G., Basheti, I., & Al-Balas, Q. Compounds and pharmaceutical compositions for treating coronavirus disease and methods of preparation thereof. WO2023238167 Worldwide, December 14, 2023. C07. Due to limited funds and the fact that the primary authors had to confirm the concept in affiliated research laboratories, made the possibility of actually performing multiple experiments very difficult. Nevertheless, confirming that carnosine and LMS1007 both have breast cancer and COVID-19 activities in the same ACE2 inhibition range adds more robustness to the findings of this study.

## Ethical statements

The current study did not include an in vivo or human experiment.

## Data availability statement

Data associated with the current study has never been deposited into a publicly available repository. Data will be made available on request.

## CRediT authorship contribution statement

**Sarah A. Melhem:** Resources, Methodology, Investigation. **Loai M. Saadah:** Writing – review & editing, Writing – original draft, Visualization, Validation, Supervision, Software, Project administration, Formal analysis, Data curation, Conceptualization. **Zeena S. Attallah:** Resources, Methodology, Investigation. **Iman A. Mansi:** Resources, Methodology, Investigation. **Saja H. Hamed:** Resources, Methodology, Investigation. **Wamidh H. Talib:** Resources, Methodology, Investigation.

## Declaration of competing interest

The authors declare that they have no known competing financial interests or personal relationships that could have appeared to influence the work reported in this paper.
